# Positron Annihilation and Complementary Studies of Copper Sandblasted with Alumina Particles at Different Pressures

**DOI:** 10.3390/ma10121343

**Published:** 2017-11-23

**Authors:** Paweł Horodek, Krzysztof Siemek, Jerzy Dryzek, Mirosław Wróbel

**Affiliations:** 1 Institute of Nuclear Physics Polish Academy of Sciences, PL-31342 Krakow, Poland; siemek.krzysztof@gmail.com (K.S.); jerzy.dryzek@ifj.edu.pl (J.D.); 2 Joint Institute for Nuclear Research, 6 Joliot Curie Str., 141980 Dubna, Russia; 3 Faculty of Metals Engineering and Industrial Computer Science, AGH University of Science and Technology, 30 Mickiewicza Ave., 90-059 Krakow, Poland; mwrobel@agh.edu.pl

**Keywords:** defects, copper, sandblasting, positron spectroscopy

## Abstract

Positron annihilation spectroscopy and complementary methods were used to detect changes induced by sandblasting of alumina particles at different pressures varying from 1 to 6 bar in pure well-annealed copper. The positron lifetime measurements revealed existence of dislocations and vacancy clusters in the adjoined surface layer. The presence of retained alumina particles in the copper at the depth below 50 µm was found in the SEM pictures and also in the annihilation line shape parameter profiles measured in the etching experiment. The profiles show us that the total depth of damaged zones induced by sandblasting of alumina particles ranges from 140 µm up to ca. 800 µm and it depends on the applied pressure. The work-hardening of the adjoined surface layer was found in the microhardness measurements at the cross-section of the sandblasted samples.

## 1. Introduction

The process of bombarding a surface with small abrasive particles is called sandblasting. It is often used in industry to clean surfaces of different objects by removing paints, impurities, or corrosion products. It is the basic tool in eliminating oxides created during heating of alloys destined to be future implants in prosthetics [1]. Another important application of sandblasting is induction of nanocrystallization [2].

The most studied materials exposed to sandblasting are dental alloys [1], stainless steels [3], titanium [4], and titanium alloys [5]. Scanning electron microscopy (SEM), atomic force microcopy (AFM), X-ray diffraction (XRD), or microhardness measurements are commonly used to investigate the morphology of sandblasted surfaces or properties of the nanocrystallized subsurface regions [6,7]. Studies revealed that sandblasted surfaces are sensitive to the stream pressure, size, and chemical composition of the applied particles.

Fast particles hitting the surface, next to changes in, e.g., roughness, generate an avalanche of stresses and plastic, as well as elastic, deformations below it. These lead to the creation of a number of different kinds of structural defects with unknown distributions and occupied depths. Studies are rarely performed in this context, although the presence of defects has a direct impact on the properties of the subsurface region [8]. Their occurrence is revealed in changes of strength, ductility, and microhardness [9,10]. Corrosion can be also connected with defects [11]. Moreover, faster wear of materials starts at the atomic level and can affect the lifetime of the material [12].

In this paper we report positron annihilation (PAS) and complementary studies of pure copper samples exposed to sandblasting of alumina particles at different pressures. Copper was chosen because it is a metal whose special physical, chemical, electrical, and mechanical properties locate it on the third position among the most commonly used metals in the world. The wide spectrum of applications and well-known structure make copper a convenient object for basic studies [13,14].

The experimental techniques were selected to provide wider discussion related to impact of sandblasting on changes generated on the surface and in the region below it. Positron annihilation spectroscopy (PAS) is the main applied method. This is a suitable tool for the detection of open-volume defects, such as vacancies, their clusters, dislocations, voids, etc. It allows us to recognize the type of introduced structural defects and their depth profile. The successful application of PAS in studies of defects in materials treated in different ways is well documented in the literature [15,16,17,18].

Although there are many studies of sandblasted copper reported in the literature, PAS investigations have not yet been done. Erosion of copper was monitored with surface profile measuring by observation of the profile produced by blasting with alumina grit particles 30 µm in diameter [19]. It was demonstrated that erosion depends on incident angle of the sand stream and increases from the minimum for normal incidence to the maximum for angles in the range between 10° and 20°. Then it decreases to zero for angles reducing to 0°. Using electron micrographs, Miller et al. [20] showed that slower time decay of Raman signals is observed due to the growth of a surface oxide layer. In turn, the initial Raman signal strengths are similar in comparison with only etched plates. Yuan et al. [21] studied the effect of Cu surface roughening induced by sandblasting on the oxidation behaviour of Cu. They demonstrated that increasing the sandblasting treatment time reduces the oxide film growth and suppresses the oxide film delamination from the Cu substrate.

We intend to characterize surface and subsurface layer of sandblasted copper depending on the pressure of air transferring alumina particles. The main focus is on a demonstration of the depth profiles of structural defects concentration and evaluation of their kind/size. Additionally, SEM pictures, microhardness, and roughness parameters were also tested to supplement the studies.

## 2. Materials and Methods

### 2.1. Sample Preparation

**Figure 1:**
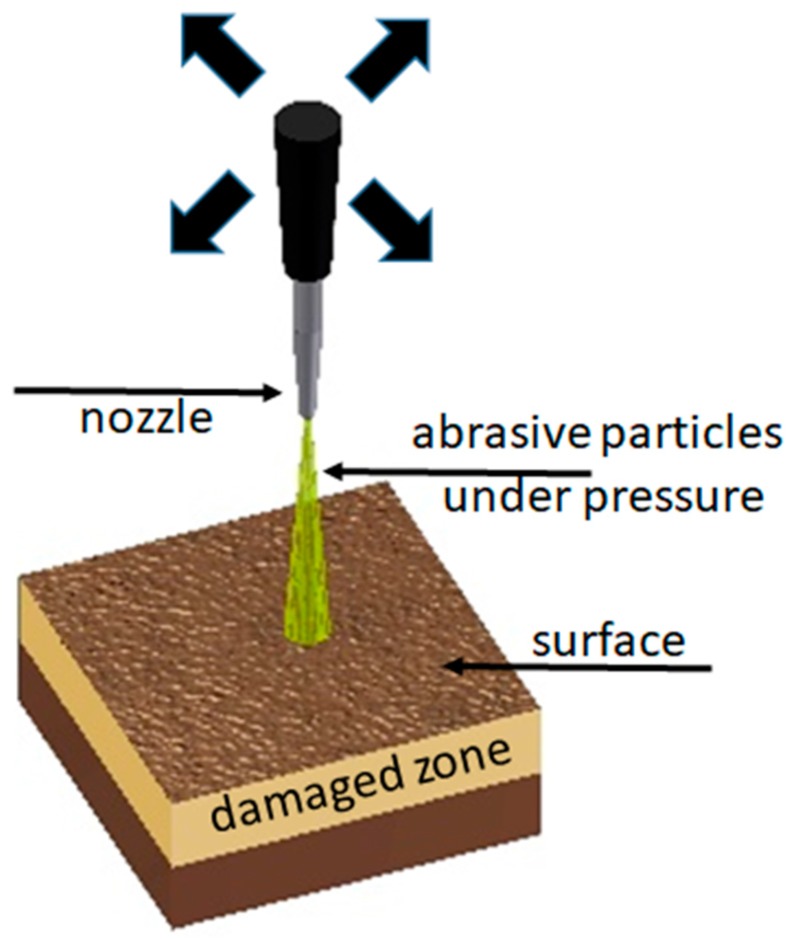
The scheme of the sandblasting procedure of the studied samples.

Commercial purity copper (99.95 wt %), produced by KGHM Polska Miedz, Warsaw, Poland and purchased from KBH Akord, Krakow, Poland, was the object of studies. All samples with dimensions of 10 mm × 10 mm × 5 mm were firstly polished to obtain maximally smooth surfaces. Then they were annealed at 1000 °C for 4 h in a vacuum of 10^−5^ Torr and slowly cooled to room temperature. This procedure allowed us to prepare identical specimens, with only residual defects, in conditions protected from oxidation. Two samples were saved as reference, while others were exposed to surface treatment. Sandblasting was performed using a Renfert Vario Basic Jet blaster, Hilzingen, Germany. The abrasive material Edelkorund containing 99.8% aluminum oxide (Al_2_O_3_) with a size of 250 μm was applied. The surfaces were blasted for 60 s under pressures of 1–6 bar (with 1 bar steps) with a distance of 10 mm between the sample and the perpendicularly-directed nozzle. The nozzle size was 1 mm. There were always two specimens prepared for each employed pressure to use in positron measurements. The scheme of the sandblasting procedure is shown in Figure 1.

### 2.2. Positron Measurements

The positron lifetime (LT) measurements were performed at Institute of Nuclear Physics Polish Academy of Sciences in Krakow using a fast-fast spectrometer based on BaF_2_ scintillators. The timing resolution equaled ca. 250 ps. The isotope ^22^Na with an activity of 32 µCi enveloped into two 7 µm thick kapton foils was placed between two identical samples. The analysis of obtained spectra, including 10^6^ counts, was provided with the LT program [22]. The source contribution, background, and finite time resolution were taken into account as adjustable parameters in the deconvolution procedure.

Doppler broadening of the annihilation line (DB) measurements were performed at the Joint Institute for Nuclear Research in Dubna using an encapsulated ^22^Na positron source with an activity of 15 µCi. The isotope was closed in a copper capsule of 5 mm in diameter and 7 µm thick titanium window. The exit of positrons was only possible through the window according to this geometry. The source was placed in front of the detector with the window directed to the top. The sample covered the window with the studied surface. Positrons were implanted in all directions giving information from the sample and the capsule. Any changes in PAS characteristics could come only from the sample. More details of the measurement setup are given in [23]. The DB experiments were conducted using an HPGe detector with an energy resolution of 1.20 keV for an energy of 511 keV. Each obtained spectrum was analyzed to calculate the annihilation line shape parameter called the S parameter. It is given as the ratio of the area below the central part of annihilation peak to the total area in the range of this line. The energy interval taken for calculation is always constant within the whole measurement session. Extraction is additionally provided without background. Usually the energy window is fixed in this way to obtain a value of the S parameter close to 0.5. The extraction of the measured annihilation lines was performed using the SP code [24] taking into account following energy window: |E_γ_ − 511 keV| < 1.38 keV. Such a window is at the annihilation line in the low-momentum electron region, and their greater number is in the open volume defects. Then, the value of the S parameter is extremely sensitive to the open volume defects which trap positrons. Briefly, the value of the S parameter increases when their concentration increases, however, the dependency is not linear and can also be sensitive to the size and type of defect (see Appendix A).

Positrons emitted directly from ^22^Na are characterized by continuous energy spectrum with the energy end-point equal to 545 keV. For this case the number of positrons decreases exponentially with the depth increase from the entrance surface. The linear absorption coefficient for ^22^Na positrons in copper is about 348 cm^−1^ [25]. Thus, about 63% of primary emitted particles annihilate at the mean implantation depth, which is about 26 μm (or 99% at the depth up to ca. 119 μm). The total depth of the damaged layer is much higher than this value, thus, obtaining the depth dependencies of the measured annihilation characteristics requires a special technique.

It has been proved in many experiments that sequential removal of layers by chemical etching and measurements of annihilation characteristics make it possible to detect the depth profile in an accurate way. Chemical etching does not produce any defects which could disturb the initial defect depth distribution [15,16]. In our studies the samples were etched in a 25% solution of nitride acid and distilled water. A thin layer of about 10 µm was carefully removed by this mixture. The thickness of the sample was measured using a digital micro-screw with ±2 µm accuracy.

### 2.3. Surface Characterization

Scanning electron microscopy (SEM) tests were performed at AGH University of Science and Technology in Krakow using Hitachi S-3500N SEM (by Hitachi Ltd. Tokyo, Japan), equipped with EDS Noran 986B-1SPS (by Thermo Scientific, Waltham, MA, USA). The roughness was determined from optical profilers obtained by WYKO NT9300 (by Veeco Instruments, Plainview, NY, USA). The roughness parameters were verified in four independent measurements for each sample. The chosen area was located close to the center and covered 4 mm^2^ in a single test. Any window filtering option for obtaining values was not applied during the tests.

### 2.4. Microhardness Tests

Samples sandblasted at 1, 3, and 6 bar were chosen as the representative ones for the measurement of the microhardness depth profile. To this aim, the samples were cut using a diamond saw perpendicularly to the surface to obtain the cross-section. Then, the surface of the cross-section was simultaneously ground mechanically using fine-grained SiC sandpaper with grits up to 4000 and finally polished up. Cutting, grinding, and polishing were conducted according to a procedure dedicated for tests by electron backscattered diffraction [26]. Struers’ devices and materials were used in this preparation. The microhardness measurements were carried out with TUKON™ 2500 manufactured by Wilson Instruments—An Instron Company, Norwood, MA, USA. Knoop’s method was realized with a load of 0.1 N (HK0.01). Tests abided by the recommendations of the ISO 4545 standard.

**Figure 2:**
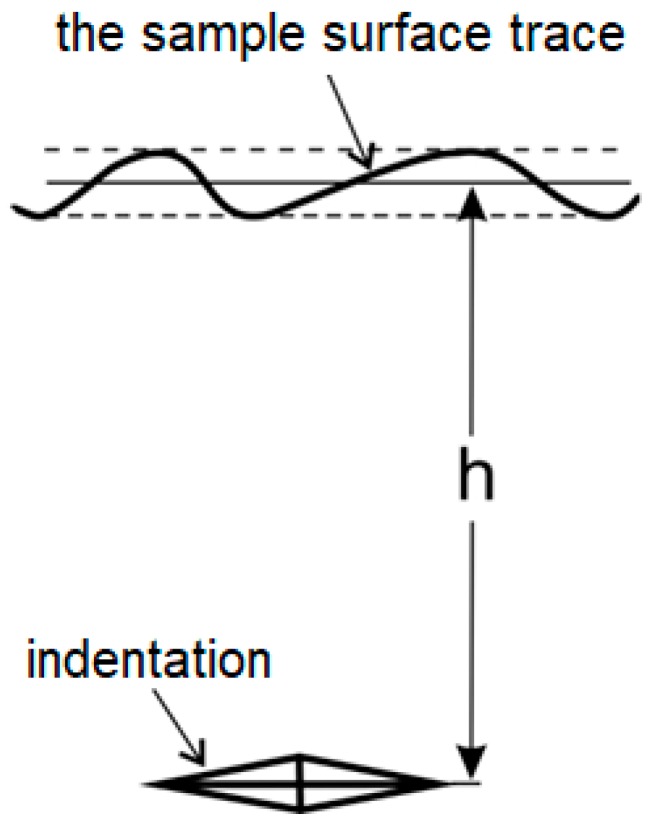
A schematic illustration of microhardness measurement procedure.

During measurements longer diagonal was always parallel to average trace of the blasted plane. The length of this diagonal and its distance to this shape (h in Figure 2) were measured each time after impression execution.

## 3. Results and Discussion

### 3.1. SEM and Optical Profilers

**Figure 3:**
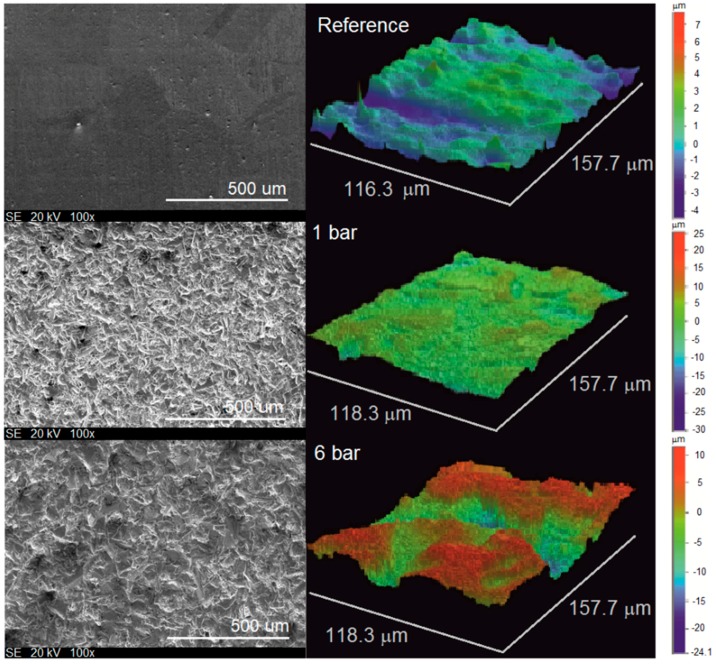
SEM and optical images of reference and sandblasted copper specimens at 1 and 6 bar.

As expected, the sandblasting definitely modifies the morphology of the surface can be seen in Figure 3. It shows the surfaces of reference and sandblasted at 1 and 6 bar samples observed by optical profilometry and SEM. It is clearly visible that the figures differ and this proves the applied pressure influences the roughness. Roughness seems to be dependent on pressure.

**Figure 4:**
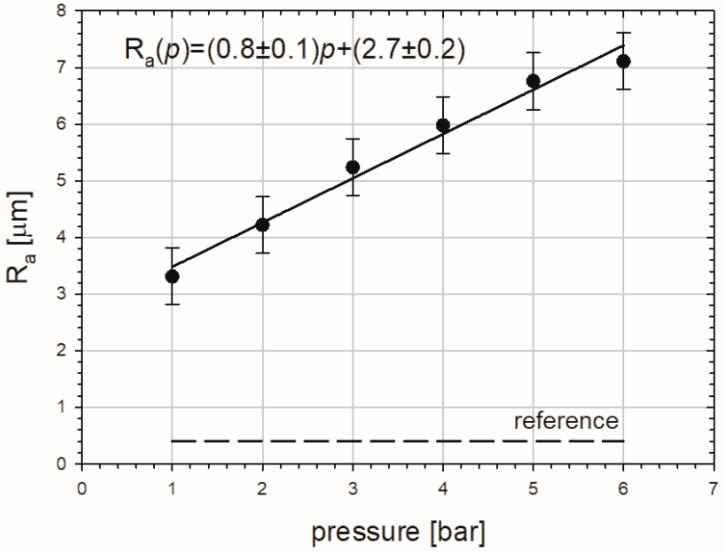
Mean roughness of the sandblasted copper specimens in dependency on the blasting pressure. The solid black line represents the linear least-squares fit to the obtained points and the results are given above, where *p* is the pressure in bar. The dashed one represents the value of *R_a_* for the reference sample.

Results of the quantitative examination of optical profilers for all studied samples were plotted in Figure 4 as the roughness average *R_a_* parameter versus pressure. *R_a_* is the mean height expressed in micrometers as calculated over the entire measured length or area. In three-dimensional case it is given as:

(1) $$R_{a}=\frac{1}{MN}\sum_{i=1}^{M}\sum_{j=1}^{N}\left|Z_{ji}\right|,$$

where *M* and *N* are number of data points in *X* and *Y*, and *Z* is the surface height relative to the mean plane. The dashed line represents the value of *R_a_* = 0.4 µm for the reference sample. In turn, the *R_a_* parameter is higher for sandblasted specimens and increases with pressure. Additionally, the linear tendency is marked in the range of applied pressures. However, deviation from this trend below 1 bar and above 6 bar cannot be excluded. The result of the least-squares fit to the obtained points is presented in Fig. 4 as the straight line.

**Figure 5:**
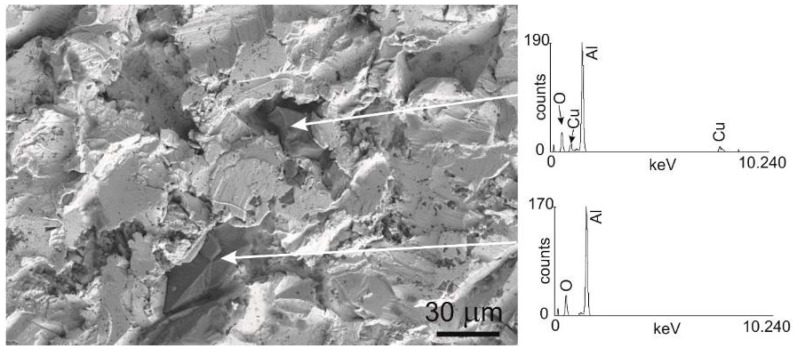
SEM image of a representative sandblasted copper sample, the arrows show the alumina particles. On the right, EDS analysis of chemical composition insets.

Presented in Figure 5, the SEM image of a representative sample additionally reveals pounded parts of impacted abrasive particles. The obtained EDS analysis of chemical composition confirms the presence of the expected components, such as Cu, Al, and O. Thus, the alumina particles remain at the surface of the copper specimens.

### 3.2. Positron Lifetime Results

**Figure 6:**
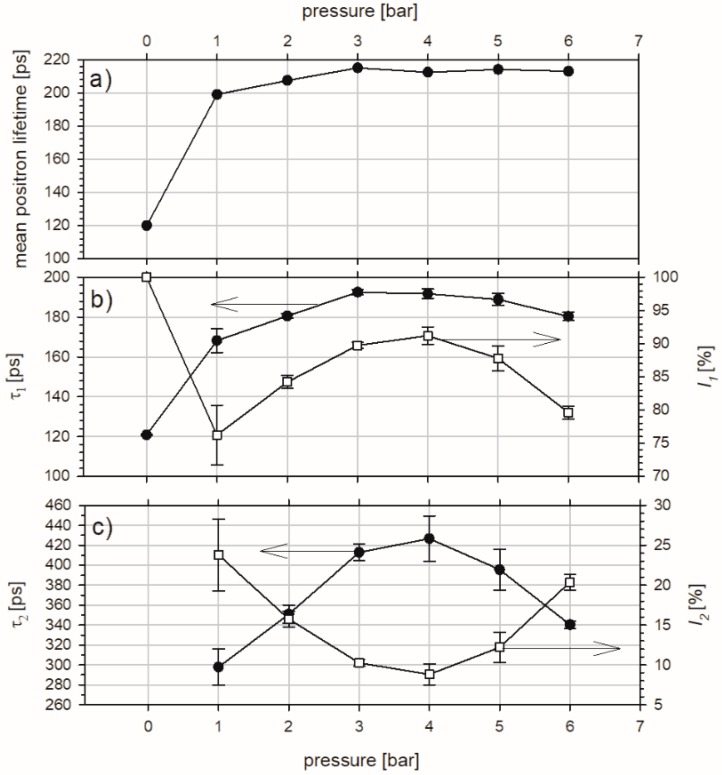
Dependencies of the mean positron lifetime $\bar{\tau }$ (**a**), the first lifetime component with its intensity (**b**), and the second lifetime component with its intensity (**c**) on pressure obtained from the positron lifetime spectra measured for the copper samples exposed to sandblasting for 1 min with alumina particles.

The positron lifetime spectra (LT) were measured directly at the surfaces of sandblasted samples, as shown in Figure 6. In this way, the gained information comes mainly from the depth of 26 µm, but slight contributions from the whole penetrated depth can also have an impact on it. Only a single lifetime component equal to 120 ± 1 ps was resolved from the LT spectrum obtained for the reference sample. It corresponds well with the positron lifetime in the well-annealed pure copper [27]. This value ensures that samples were free of defects which could have been introduced during preparation.

As it was mentioned above, the alumina particles are present in adjoined surface layer. The SEM images of the samples’ surfaces, such as those shown in Figure 5, allowed us to roughly estimate that about a 10–20% volume fraction is occupied by these particles on the basis of the stereology method [28]. Due to the differences in density between the copper matrix and alumina only about 2–5% of positrons annihilate in these particles. Foster et al., reported a value of the mean positron lifetime in Al_2_O_3_ at room temperature equal to 150 ps [29]. In turn, Noguchi et al. [30] noticed two lifetime components, e.g., *τ*_1_ = 159 ± 8 ps, *τ*_2_ = 720 ± 30 ps, *I*_2_ = 2.2% for polycrystalline Al_2_O_3_.

In our LT spectra no long lifetime component is observed, however, in the deconvolution procedure the LTC of 150 ps with an intensity of 3%, as originating from annihilation in the alumina particles, was added. Two lifetime components were also resolved in all LT spectra. We should notice that the contribution of the added component of 150 ps did not affect the deconvolution procedure, i.e., no change in the Χ^2^ value was observed, which indicates the marginal contribution of the alumina particles to the LT spectra.

In Figure 6a the mean positron lifetime $\bar{\tau }$ versus pressure is shown. It is defined as:

(2) $$\bar{\tau }=I_{1}\tau_{1}+I_{2}\tau_{2}$$

where *I*_1,2_ and *τ*_1,2_ are intensities of lifetimes respective resolved from the LT spectra and it gives information about the total changes in the defect structure in the samples. $\bar{\tau }$ increases with pressure and saturates quickly above 3 bar pressure of the alumina stream.

The value of *τ*_1_ (Figure 6b), related to the first kind of defect detected in the studied samples, varies from 168 ± 6 ps to 193 ± 1 ps with intensities *I*_1_ between 76 ± 5% and 91 ± 1%. The highest values of *τ*_1_ and *I*_1_ were noted for the pressure of the sand stream equal to 3 and 4 bar. Similar values (164 ± 10 ps and 181 ± 10 ps) were noticed by Hinode et al. [31] in copper exposed to thickness reduction of 3% and 13%. In cold-rolled copper Lynn et al. [32] observed a steady increase of the mean positron lifetime from 157 ps (for a single crystal) to 173 ps as one goes to progressively finer grain-sized material (finally 169 µm). Campillo Robles et al. [33] reported calculated positron lifetime for single vacancy in a wide range of periodic elements. In the case of copper, positron lifetime was found to depend strongly on applied model and varied from 153 ps to 200 ps. The measured value of the positron lifetime in a single vacancy defect equals 180 ps according to Schaefer et al. [34]. *τ*_1_ = 166 ± 2 ps, observed by Čížek et al. [35], in copper samples prepared by high-pressure torsion (HPT) was interpreted by the authors as positrons trapped at dislocations. Additionally, the lifetime of 164 ps was shown by McKee [36] as representing positron annihilations at dislocations. On the basis of the above discussion dislocations have been introduced in the sample sandblasted at 1 bar while, for specimens processed with higher pressure, single vacancies occur.

The value of *τ*_2_, depicted in Figure 6c, varies from 298 ± 18 ps for 1 bar to 427 ± 23 ps registered for 4 bar with intensities *I*_2_ in the range between 9 ± 1% and 23 ± 5%, respectively. This reveals the second type of defect present in the studied sandblasted samples. According to calculations for nanocrystalline Cu performed by Zhou et al. [37] *τ*_2_ values obtained by us can be interpreted as an occurrence of vacancy clusters containing from eight up to ca. 40 vacancies. Positron lifetimes in the range 290–320 ps obtained in HPT-deformed Cu by Čížek et al. [38] were similarly recognized by these authors as clusters containing 7–9 vacancies. The highest value of *τ*_2_ appears t a pressure of 4 bar, however, taking into account the variation of presented points the plateau in the range 3–5 bar is possible. The *τ*_2_ increases with pressure to a maximum in the mentioned range while, for a pressure of 6 bar, the well-marked decrease is noted. In this case the inverse proportion between *τ*_2_ and *I*_2_ is revealed. *I*_2_ declines with the pressure achieving a minimum and starts to rise. Given this, concentration of generated defects decreases with the pressure increase, but only up to 4 bar. Above this value the defect concentration increases. Consequently, the size of the vacancy clusters represented by *τ*_2_ expands up to 4 bar and reduces for higher pressures.

To sum up, two lifetime components prove the existence of two kinds of defects in each studied sandblasted sample. In the case of blasting under the pressure of 1 bar they were recognized as dislocations and clusters of about eight vacancies. For other samples, next to single vacancies, larger clusters containing upwards of 40 vacancies appeared.

### 3.3. Defect Depths Profiles

**Figure 7:**
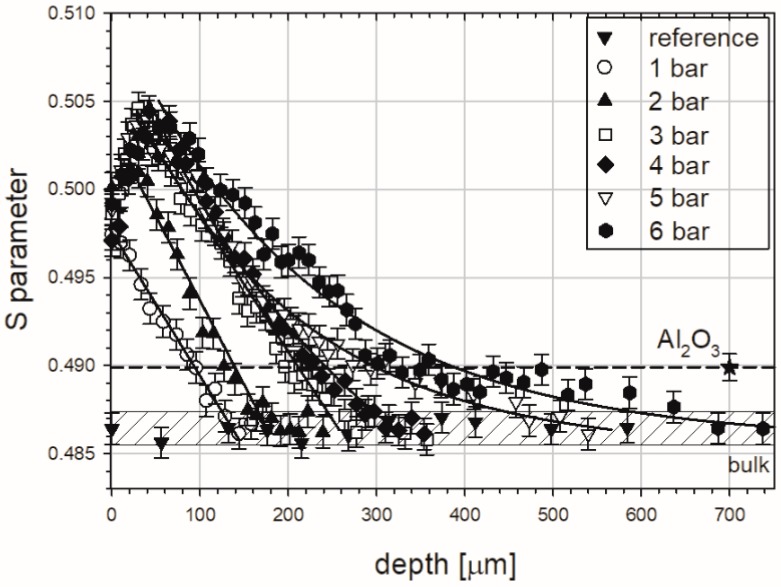
S parameter dependent on the depth for the well-annealed copper samples sandblasted with alumina particles at different pressures. The drop line marks the value of the S parameter obtained for pure alumina. The solid lines represent the best fits of linear or exponential decay functions to decreasing parts of measured profiles.

LT spectroscopy is the most effective PAS technique, however, it is time-consuming. For instance, 12 h were needed to obtain only one LT spectrum with a satisfying statistic. For this reason the defect depth profiles, requiring multiple measurements along with sequenced etching, were determined using DB spectrometry. This approach allowed us to reflect the shape of mentioned distributions precisely, what is visible in Figure 7 as the S parameter versus etched depth.

The hatched region tags in Figure 7 represent the value of the S parameter for a reference, well-annealed sample, i.e., for the “bulk”, where positrons annihilate in the delocalized state only (certainly, after annealing, some residual defects remained, but they are neglected). We expect that this value will be reached at a depth where the interaction of processes at the surface during blasting fade out. The measured points in Figure 7 are represented by black triangles. In this case constant values of the S parameter shows us that sequenced etching is neutral for our measurement procedure.

The different shape and colour points represent the S parameter values for samples sandblasted at different pressures, as shown in Figure 7. Presented profiles are placed above the bulk region pointing to the existence of the layer below the surface occupied by defects introduced by the sandblasting. The close surface region is characterized by the S parameter increasing with depth. This feature is clearly visible for samples treated at a pressure range of 2–6 bar. Then, after achieving the maximum the S parameter decreases towards the value characteristic of the bulk region pointing to the end of the damaged zone. The unusual shape of the obtained profiles and connections between them should be highlighted. We supposed rather that the S parameter will be decreased with depth with a given type of tendency saved for all samples. For this reason, discussion of a few aspects is required in their analysis.

**Figure 8:**
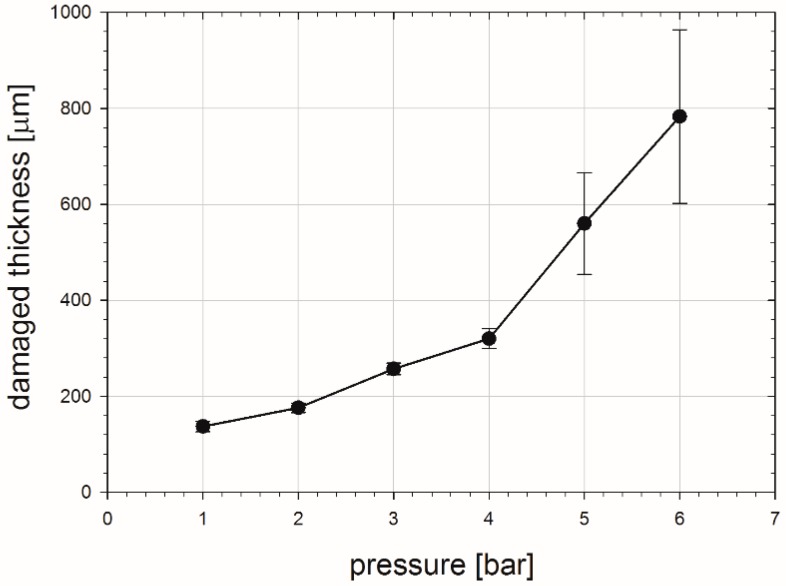
Total thickness of the damaged zones obtained from PAS measurements dependent on the pressure applied in the sandblasting of copper.

The characteristic decay of the S parameter values above ca. 50 µm is well understood. With the depth increase fewer defects are generated during sandblasting and their concentration is the highest at the surface. The total depth of the damaged zone is extended from 140 to 780 µm and strictly depends on the applied pressure. This is shown in Figure 8.

**Figure 9:**
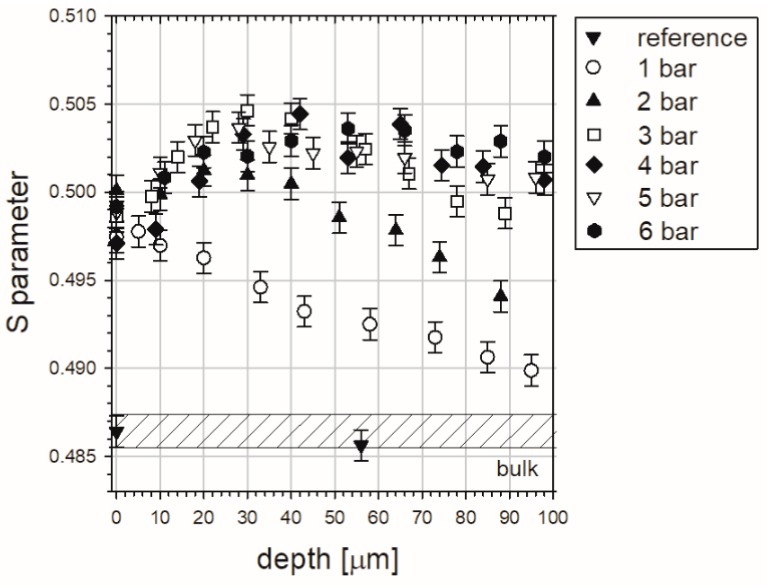
S parameter dependent on the depth in the range 0–100 µm for the well-annealed copper samples sandblasted with alumina particles at different pressures.

The maximum, at the depth of about 50 µm (Figure 7), is a new interesting feature which was not observed in distributions registered for copper exposed to other treatment processes [16,17]. For analysis of the near surface region the depth up to 100 µm was additionally presented in Figure 9. On closer inspection this maximum also depends on the pressure, and it ranged from 20 to 50 µm for the increasing pressure. We accept the explanation that the alumina particles which are pushed into the samples are responsible for this maximum. The S parameter value for the alumina, measured in independent experiments, is higher in comparison with its value obtained for the pure well-annealed copper, but lower than the S parameter value for the deeply-deformed copper, after 80% thickness reduction. Thus, positrons which are implanted into the sandblasted copper sample with pushed alumina particles can annihilate in deformed copper, but also in these particles. Finally, the measured S parameter, which is averaged over all possible annihilation states, can be lower than its value for the deformed copper. The alumina particles are present exclusively in the layer ca. 50 µm below the level where only deformed copper is extended.

Solid lines in Figure 7 represent the best fits of the linear and exponential decay functions to the parts of profiles in the regions between maximum and the end. In case of distributions representing blasting at 1–3 bar, the linear tendency was observed. In turn, the exponential character can be assigned to the rest of profiles. The former tendency was registered in machined copper [16], while the latter was noted in copper samples exposed to sliding and cutting [17]. Various types of S parameter distributions from one treatment process were also obtained for austenitic stainless steel exposed to sliding under varied dry load conditions [39].

### 3.4. Microhardness Profiles

The fact that the existence of structural defects has a significant impact on hardening of materials has been repeatedly reported in the literature [40].

**Figure 10:**
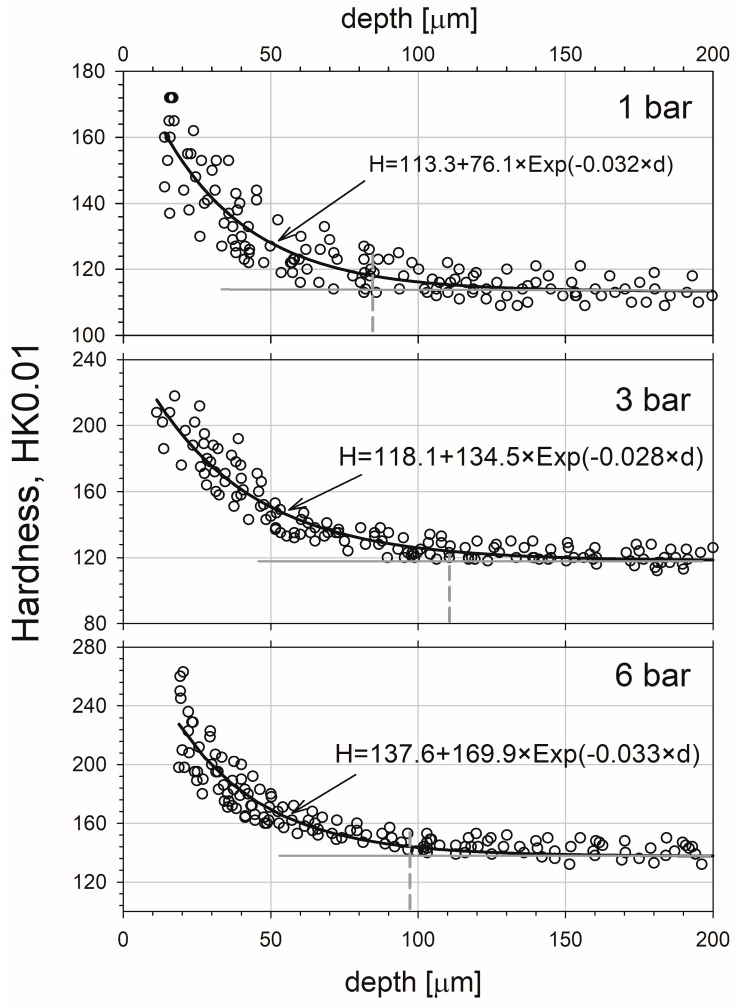
The depth profile of microhardness of the studied copper samples exposed to sandblasting at various pressures. The black solid lines represent the best fits of the exponential decay functions to the experimental points. The dashed vertical lines tag the depth of the workhardening zone for each sample. The error in the hardness measurement can be roughly estimated as ca. 20 HK for sample blasted at 1 bar and ca. 40 HK for those blasted at 3 and 6 bar.

In Figure 10 the microhardness profiles for the sample sandblasted at 1, 3, and 6 bar were depicted. One can notice a significant increase of microhardness in the layer adjoining the surface in comparison to the bulk region at the depth above 150 µm. For the lowest pressure the increase is from ca. 110 to 170 HK0.01, but for the highest it is from ca. 130 to 260 HK0.01. This increase can by related mainly to the matrix strain hardening due sandblasting process because only hardness results obtained from the copper matrix were included in the chart. It is not caused by the presence of the alumina particles, which are much harder. Alumina hardness is in the range of 800–2000 HV [41], which gives a range of 822–1800 HK. With the depth increase, the microhardness decreases quickly, and for all cases the exponential decay character is noted.

The best fits of exponential decay functions are presented with the solid lines shown and the adjustable parameters are quoted in Figure 7. The solid horizontal lines shown in Figure 7 represent the saturations of obtained profiles. The square of the regression coefficient for analytical fitting functions is equal 0.86, 0.91 and 0.93 for 1, 3, and 6 bar, respectively. The dashed vertical ones mark the depth of 5% increase in the average hardness. This was taken as the total depth of strengthened zone, and it is about 80, 110, and 100 µm for samples blasted at 1, 3, and 6 bar, respectively. Thus, the effect is more or less the same between 3 and 6 bar pressures. However, the HK0.01 hardness at the adjoined the surface zone strongly depends on the pressure, and it is ca. 190, 250, and 310 HK0.01 for 1, 3, and 6 bar, respectively (all these data were obtained from the analytical fitted functions). A similar discrepancy between the thickness of modified zone obtained from PAS and hardness measurements was found in stainless steel [39] and aluminium alloys [42] exposed to sliding. The macroscopic character of the hardness tests may be the reason of this difference. The gage volume of the single hardness measurement is relatively high and it can be the source of some errors. Additionally the sensitivity of PAS techniques is much above the sensitivity of microhardness.

One should add that the lack of the maximum at the depth of 50 µm, seen in Figure 7, is due to fact the indenter was located only in the copper matrix, not in the alumina particles and in the matrix directly adjacent to the particles.

## 4. Conclusions

The paper reports positron annihilation and complementary studies of copper exposed to sandblasting with different pressure. It was found that pressure has significant impact on roughness, hardness, kind/size of defects, and their distributions, including the range below the surface. The linear dependency between the mean surface roughness and applied pressure characterized sandblasted copper. Dislocations or single vacancies and vacancy clusters containing from eight up to ca. 40 vacancies were introduced during sandblasting. Unusual defect concentration profiles disclosed the existence of adjoined layers created by residues of alumina particles. According to this and SEM we can state that these particles are pushed to a depth of about 50 µm from the surface. The total thickness of damaged zones evaluated from PAS measurements is in the range from ca. 140 µm up to ca. 800 µm. The sandblasting causes an increase in the microhardness only at depths below 100 µm. The exponential decay of the microhardness profile was detected.

## Appendix A

According to the positron trapping model, S parameter can be expressed in the following form:

(A1) $$S=\frac{\lambda_{f}\cdot S_{f}+K\cdot S_{w}}{\lambda_{f}+K}$$

where *λ_f_* is an annihilation rate in free state, and *S_f_*, *S_w_* are values of S parameter associated with positrons annihilating in free state and trapped in defect, respectively. $K=\mu \cdot c_{w}$ represents the trapping rate, where µ is the trapping coefficient, specific for a given type of defect, and *c_w_* is the defect concentration. In this way the S parameter is the function of *c_w_* and any change of the defect concentration should modify its value. Consequently, the sensitivity of the DB technique for detection, e.g., of vacancies, depends on the trapping rate [43]. Its value for metals is large enough (ca. 10^14^ s^−1^) to detect vacancies with a concentration on the level of 10^−7^.
